# An Effective and Novel Neural Network Ensemble for Shift Pattern Detection in Control Charts

**DOI:** 10.1155/2015/939248

**Published:** 2015-08-03

**Authors:** Mahmoud Barghash

**Affiliations:** IE Department, The University of Jordan, Amman 11942, Jordan

## Abstract

Pattern recognition in control charts is critical to make a balance between discovering faults as early as possible and reducing the number of false alarms. This work is devoted to designing a multistage neural network ensemble that achieves this balance which reduces rework and scrape without reducing productivity. The ensemble under focus is composed of a series of neural network stages and a series of decision points. Initially, this work compared using multidecision points and single-decision point on the performance of the ANN which showed that multidecision points are highly preferable to single-decision points. This work also tested the effect of population percentages on the ANN and used this to optimize the ANN's performance. Also this work used optimized and nonoptimized ANNs in an ensemble and proved that using nonoptimized ANN may reduce the performance of the ensemble. The ensemble that used only optimized ANNs has improved performance over individual ANNs and three-sigma level rule. In that respect using the designed ensemble can help in reducing the number of false stops and increasing productivity. It also can be used to discover even small shifts in the mean as early as possible.

## 1. Introduction

**Figure 1:**
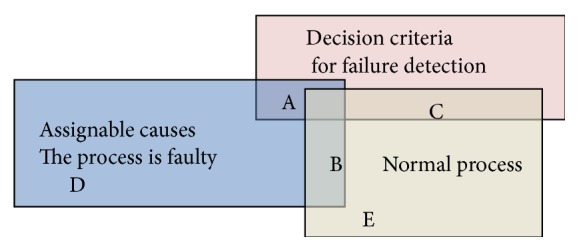
Mapping decision criteria for failure against normal and faulty process signals.

Pattern recognition in control charts is extremely important for increasing productivity and for better customer satisfaction. Manufacturing process is of random nature; however certain aspects are not random such as broken heater, worn bearings, unacceptable raw materials, worn coils, higher than needed temperature, or other process parameters. These are called assignable causes. Assignable causes (real process uncontrollable changes) can affect the process quality and must be discovered as early as possible while unassignable random causes (normal process variations) must be ignored. In case the process is allowed to continue working under assignable causes effect, quality deteriorates and more defects or defectives are produced. This case is called type II error. If the process is stopped when the process is still under normal variation (this is a false alarm) then the productivity is reduced. This is called type I error. Both errors are current in practice. Successful process control is a technique that minimizes both errors. There is usually a compromise between these two errors; that is, if we try to reduce type II error, that is, to make sure that more nonnormal causes are discovered, then more normal variations are mistaken as nonnormal and type I error is increased. If we try to reduce type I error, that is, to make sure that less normal variations are mistakenly classified as assignable, then more assignable causes are undiscovered and type II error is increased. Early discovery of faults is a subject of a large sum of current research. This is usually combined with online monitoring when this is related to manufacturing processes. Some examples of this failure monitoring are induction motor failure discovery using current signal [1], bearing fault detection [2], metal processing sensor fault diagnostics [3], and so forth. In all, the decision should be made of whether the process is subject to real change (assignable) or whether the process is still under normal causes.  Figure 1 maps the generated patterns from normal and faulty processes against the decision criterion for failure. Area B shows patterns that can be generated by both faulty and normal processes and represents a challenge for the researchers. Areas A and E are successfully classified as faulty (A) when the process is faulty and not faulty (E) when it is not faulty. Areas D and C are the main decision mistakes leading to type II error (D) whereby the process is classified as normal while the process is actually faulty and type I error whereby the process is classified as faulty while it is actually normal.

The main objective of statistical process control is to develop techniques that can be used to reduce type I and type II errors. The early attempts were based on heuristic rules of thumb. Control charts were proposed by Shewart in the early 1920s which was improved through heuristics and zone testing by Western Electric rules in 1958 [4]. Page in 1958 had developed the CUmulative SUM (CUSUM) control chart and Roberts in 1959 had proposed the Exponentially Weighted Moving Average (EWMA) control charts to increase the sensitivity to discover even small changes in the processes. Also Lucas in 1973 developed a V-mask for the CUSUM control chart to improve its abilities. The sole purpose of these traditional tools is to improve the ability to discover changes in the process. However, while using these tools, the compromise between type I and type II errors still holds. This is the reason why many researchers turned to Artificial Intelligence [5]. Several research works have been conducted in pattern recognition in control charts using Artificial Intelligence including Artificial Neural Networks (ANN) [6–12], support vector machine (SVM) [13], rule-based [14], expert system [15, 16], neurofuzzy [17], multistage neural networks [18], feature selection [19, 20], fuzzy [21], ART [22], learning vector quantization [23], neural expert [24], ensemble based techniques [5], and efficient features combined with optimized ANN [25].

Recently ensemble based techniques have seized more attention by researchers. Several research works have shown improved results when using ensemble based techniques. Ensemble based techniques identified both mean and variance shifts [26], autoregressive coefficient-invariant control chart pattern recognition model for online recognition of seven typical types of unnatural control chart patterns [27], both raw data and statistical features extracted combined with an ANN ensemble, which are used for the recognition which provides for better performance [28], a selective NN ensemble of selected NNs to classify source(s) of out-of-control signals in multivariate processes [29], identifying the source(s) of process mean shifts in multivariate control charts using an improved particle swarm optimisation with simulated annealing-based selective multiclass support vector machines ensemble [30–32] and diagnosing and classifying out-of-control signals in a bivariate process using self-organization map neural network [33], and percent correct classification for a committee of specialized recognizers [34]. While previous literature work concentrated on developing new Artificial Intelligence techniques or training algorithms, this research work focused on optimizing the performance and stability of the ensemble construction to achieve low false alarms and quick discovery of even the smallest shift in the process mean. This research work shows that different ensemble construction can give highly different results and thus emphasized the need for ensemble tuning or optimization.

**Figure 2:**
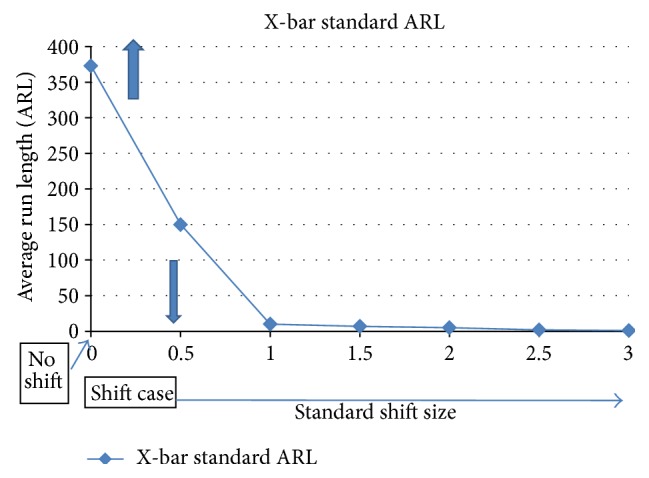
Traditional 3-sigma level ARL curve which serves as a benchmark for comparison.

Shift patterns are some of the most common assignable cause patterns. These occur when the process mean changes due to assignable causes. This is usually reflected in product characteristic changes such as size, shape, or other quality parameters. A usual measure for the success of SPC system is the average run length where the number of signal samples before an alarm is sounded and the process is out of control.  Figure 2 shows a sample ARL chart which is generated based on the basic benchmark traditional X-bar. The *x*-axis is the shift size. There is no shift at the origin or point zero. When the shift size is small, close to the origin, there will be some mix-up between shift and no shift case, while at large shift case, far from the origin, there is usually no mix-up with no shift case. The ARL at zero shift size is close to 373; that is, on average, a false alarm is issued every 373 data points. For the case of small shift 0.5 standardized shift (the system mistakenly classifies this as no shift for a large number of samples), nearly 150 data points are passed before the shift is discovered. A better performing system may reduce the number of false alarms while keeping the 0.5 standard shift at 150, or keeping the ARL at zero shift to 373 while reducing the ARL for 0.5 sigma to less than 150, or it can increase the ARL at zero shift and reduces the ARL at 0.5 sigma to less than 150.

This work is a based on previous work (Barghash and Santarisi [35] and Barghash [5]) which showed that extraordinary balance between type I and type II error can be achieved through the use of optimized ANN ensembles. This work shows that compromise between type I and type II error can be greatly improved through proper design of the population percentages and using ensembles. This work also tests the effect of the population of percentages on type I and type II errors in an attempt to optimize the ANN. This work also tests the use of optimized and nonoptimized ANN for the purpose of detecting the shift pattern which is one of the most common assignable causes in the industry.

## 2. Artificial Neural Network Ensemble

### 2.1. Artificial Neural Network Training

A neural network is composed of several interconnected neurons with the following activation function for each neuron [36]:

(1) $$\begin{array}{c} \text{output}=\varphi \left(\;\overset{p}{\underset{i=1}{\sum }}w_{i}x_{i}-\theta \;\right), \end{array}$$

where *φ* is the neuron function, *w*_*i*_ is the neuron *i*th weight, *x*_*i*_ is the *i*th input to the neuron, *θ* is the constant threshold value, and *p* is the number of inputs to the neuron.

**Figure 3:**
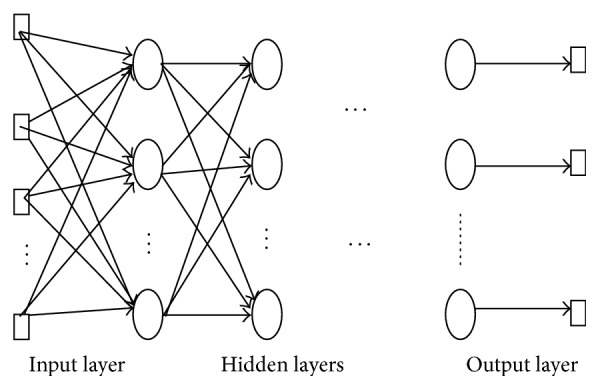
Layers of neural networks [15].

Neural networks have high interconnectivity, where all the neurons are arranged in layers. The outputs of the first layer are the inputs for the second layer as shown in Figure 3. The first layer is the input layer and the final layer is output layer. The layers in between are called hidden layers [6].

Training of the neural network is done using backpropagation where the error value and the local gradient of the error are used to estimate the new value of the weights and biases:

(2) $$\begin{array}{c} \Delta w_{ji}\left(\;n\;\right)=\eta \delta_{j}\left(\;n\;\right)y_{i}\left(\;n\;\right), \end{array}$$

where *w*_*ji*_ is the synaptic weight connecting the output of neuron *i* to the input of neuron *j*. *η* is the learning rate. *δ*_*j*_(*n*) is the local gradient for the error in output neuron *j* at iteration *n*. *y*_*i*_(*n*) is the output of the network at neuron *i* at iteration *n*. *n* is the iteration number [15].

Several types of neuron activation functions from MathWorks Matlab 7.0 were used including the Logsig sigmoid function, Tansig, and Purelin:

(3) Logsigx=11+e−x,Purelinx=b+x,Tansigx=sign⁡21+e−2x−1.

### 2.2. Artificial Neural Network Ensemble

**Figure 4:**
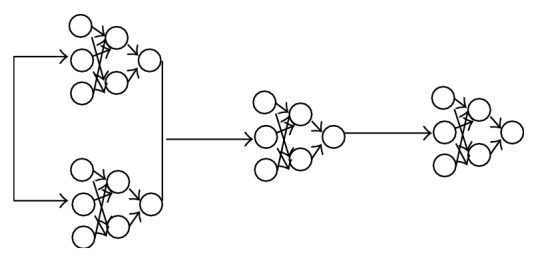
An ANN ensemble.

Figure 4 shows an ANN ensemble which is a series of ANNs. These ANNs are trained sequentially where the second layer ANN is trained using the output of the first ANN layers and so on.

## 3. Pattern Generation

**Figure 5:**
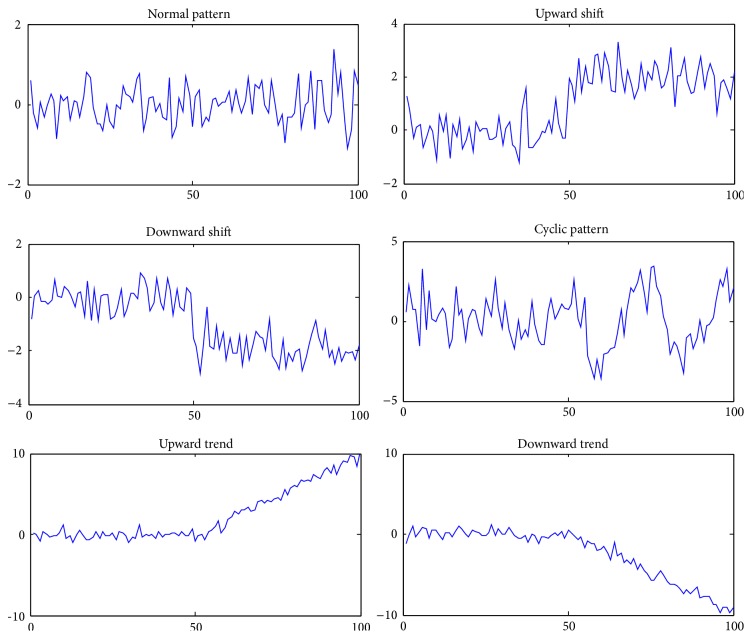
Sample patterns.

Figure 5 shows some sample patterns that are usual for the training of ANNs in pattern recognition in control charts, namely; normal (no assignable cause), shifts of both types upward and downward, cyclic, and trend of both types upward and downward.

Equation (4) is used to generate the no shift and the shift patterns:

(4) $$\begin{array}{c} \text{Generated pattern }y\left(\;i\;\right)=\mu +n\left(\;t\;\right)+d\left(\;t\;\right), \end{array}$$

where *μ* is the process mean. *n*(*t*) is the normal cause of variations which is *N*(0, *σ*). *d*(*t*) is the special cause of variations.

For the case of a shift *d*(*t*) = *u∗s*, where *u* is the position of the shift (it can be either 1 or 0) and *s* is the value of the shift in the mean.

The following standardization was chosen for the patterns:

(5) $$\begin{array}{c} Y\left(\;i\;\right)=\frac{y\left(i\right)-\mu }{\sigma_{\overset{-}{x}}}. \end{array}$$

The final variable *Y*(*i*) is a standardized variable with mean zero and a standard deviation of one for the no shift case. The mean shift values (*s*) can be expressed in a standardized form: as 4, −3, −2, −1, −0.75, −0.5, −0.25, 0, 0.25, 0.5, 0.75, 1, 2, 3, and 4 or they can be expressed as a multiple of the standard deviation. The *s* variable is set to zero for the no shift case.

## 4. Methodology

**Figure 6:**
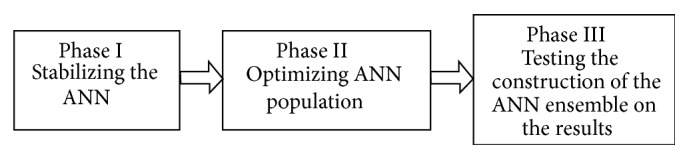
The three-phase methodology.

Figure 6 shows the proposed methodology. In this work we initially tested the effect of population percentage on the ANN performance and then tested the effect of the ensemble construction on performance.

The basic objective is to reduce both type I error and type II error. For that purpose, the average run length (ARL) is selected as it can reflect type I and type II errors in one chart at various shift levels. ARL also can be easily interpreted economically. The ARL represents the average number of points before a declaration of a nonnormal pattern. If the process is normal without a shift ideally it should go to infinity before being stopped. If the pattern is coming from a shifted process then the ARL should ideally be very close to zero.

**Figure 7:**
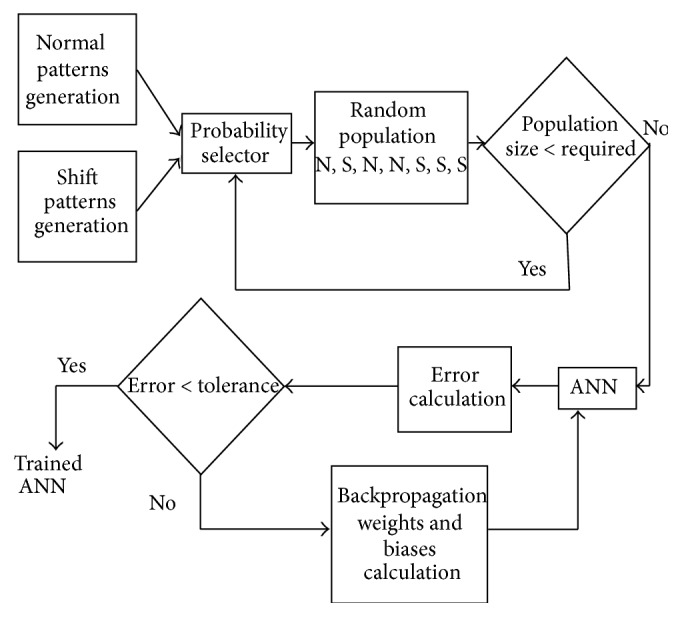
Population formation based on the shift pattern and normal pattern percentages.

Preparation of an individual neural network based on the work of Barghash and Santarisi [6] is done by generating shift patterns and normal patterns as shown in Figure 7. A randomized selector is used to perform the pattern selection to construct a population with preset pattern type percentages.

## 5. Results and Discussion

The results section considers three main phases or objectives: firstly to test the effect of multidecision points on the performance of the ANN (ANN stabilization). The second objective is devoted to testing the effect of the population percentages on the ANN ability to discover shift patterns even of the smallest size. The third objective is dedicated to testing the effect of using optimized and nonoptimized ANNs in an ensemble to discover patterns in control chart.

### 5.1. Phase 1: Stabilization of the ANN Behavior

**Figure 8:**
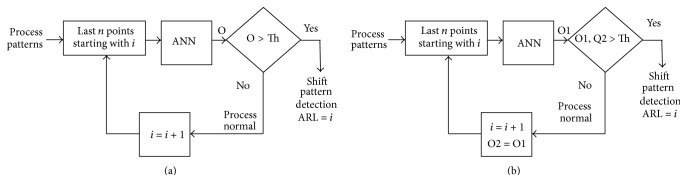
Stabilized ANN through consecutive decision points.

**Figure 9:**
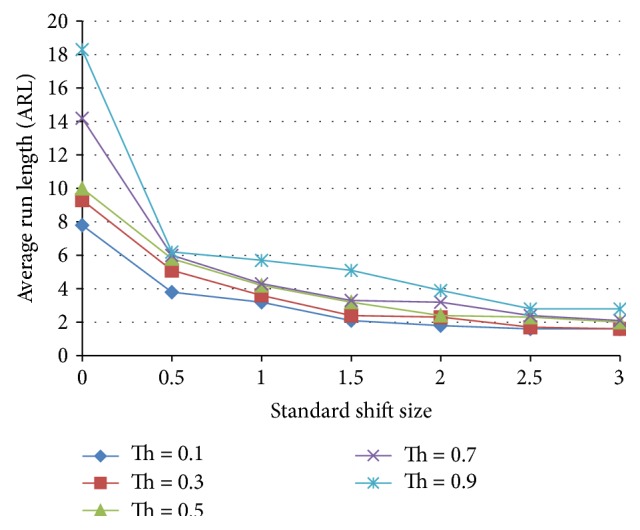
Unstabilized ANN, one decision point, of a standardized shift size for a neural network trained with 25% shift, 75% normal population.

**Figure 10:**
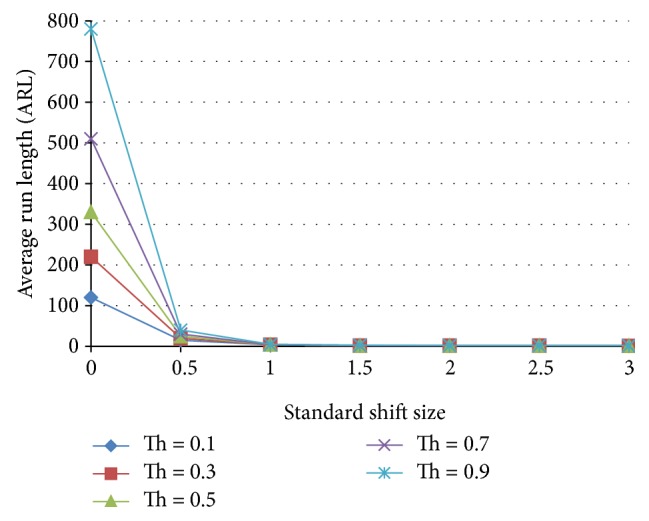
Stabilized ANN through two decision points on the average run length as a function of the standardized shift size for a neural network trained with 25% shift, 75% normal population.

Neural networks suffer from instabilities when using individual decisions. This instability can be defined as giving an erroneous decision temporarily and then giving the right decision back. This temporary misjudgment by the neural network reduces the ARL appreciably and deteriorates the performance of the neural network. This is solved by adding one or more decision points. If the ANN still insists on its decision in two or more following decisions points, this can be a sign on the existence of a nonnormal behavior. Multidecision point stabilization is tested in accordance with Figure 8, where the case of a single decision and 2 consecutive decision points are tested and the results for these two cases are shown in Figures 9 and 10, respectively. The performance of both ANNs is tested at triggering threshold levels 0.1–0.9. Two clear conclusions can be drawn; firstly, the threshold is a tuning parameter for the performance of the ANN.

And secondly, the unstabilised ANN is far worse than stabilized ANN in terms of the number of false alarms. Thus for the rest of this work, only stabilized ANNs are considered.

### 5.2. The Effect of Population Percentages

**Figure 11:**
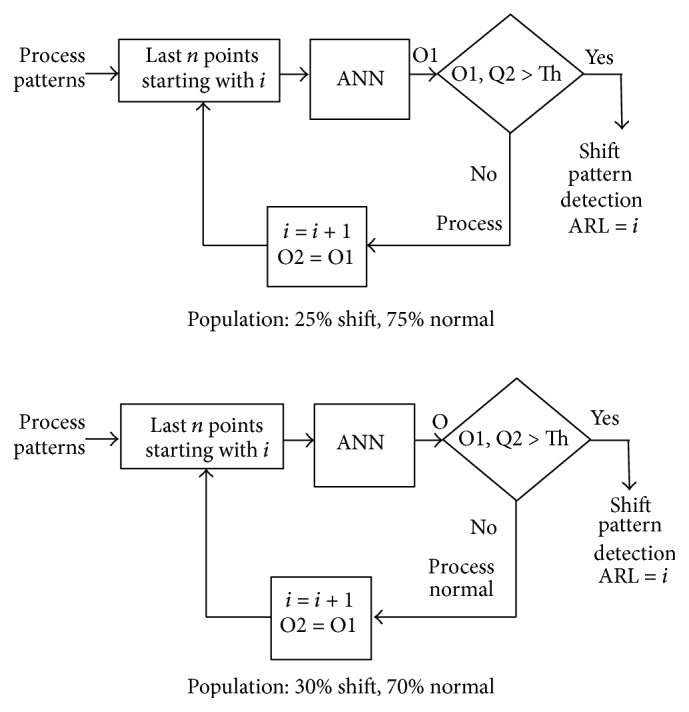
Population percentage determination for the case of individual ANNs.

**Figure 12:**
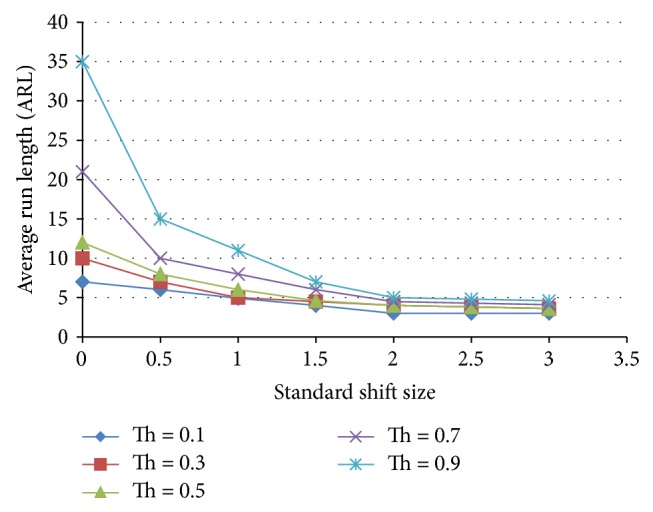
Effect of threshold on the average run length as a function of the standardized shift size for a neural network trained with 70% shift, 30% normal population.

**Figure 13:**
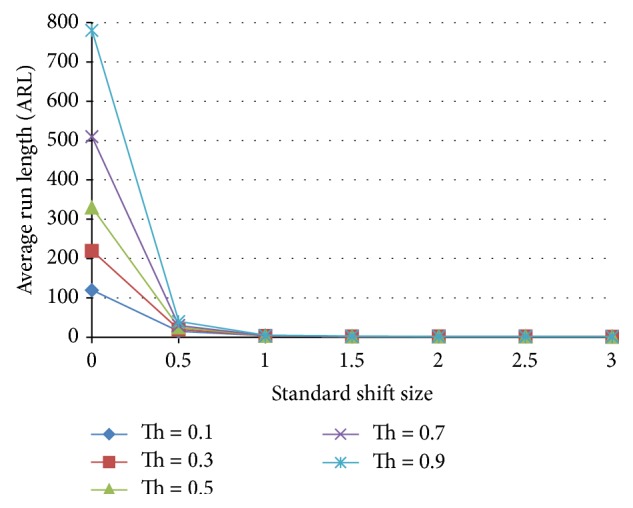
Effect of threshold on the average run length as a function of the standardized shift size for a neural network trained with 25% shift, 75% normal population.

The effect of population percentages is analyzed according to Figure 11 where the ANNs are trained in accordance with different population percentages tested for the ARL. Figures 12 and 13 show the results of a nonoptimized 30% normal and 70% shift pattern and the optimized ANN trained with 75% normal and 25% shift pattern. The nonoptimized ANN ARL is worse than that of the benchmark X-bar chart with three-sigma level rule. For the case of optimised ANN, the ARL for the case zero shift is extremely high while reducing the ARL for the case of standard 0.5 shift level to less than 150.

### 5.3. Ensemble Results

**Figure 14:**
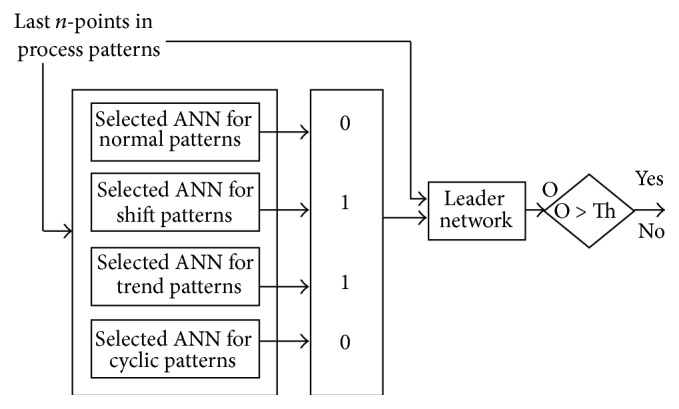
Suggested ensemble construction.

The basic shape of the generalized suggested ensemble is shown in Figure 14. The first stage is a selected neural network from four main categories: normal pattern detectors, shift pattern detectors, trend pattern detectors, and cyclic pattern detectors. Then the last two decisions of each neural network are passed to the leader net which is trained to give the decision of whether the pattern is normal or not. A question may arise on the reason for considering patterns other than normal and shift patterns in this work, although only shifts are intended to be discovered. The reason has two sides; the first relates to the fact that these patterns are present in real life and in other research works. Thus, it is imperative that the suggested ensembles be designed to discover and classify all patterns, although this work reports only on its ability to discover shift patterns. The second side relates to the problem that the ability to discover shift patterns might be affected if more patterns are included; thus it seems more logical to include these in any realistic attempt to recognize shift patterns in control charts.

#### 5.3.1. Ensemble 1 Results

**Figure 15:**
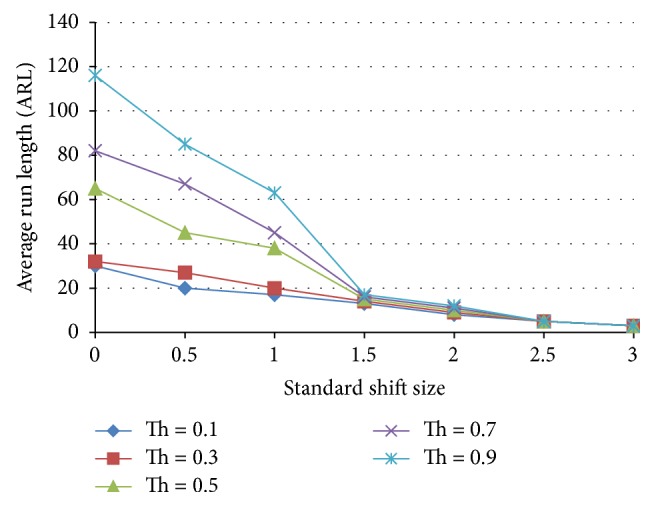
Effect of threshold on the average run length as a function of the standardized shift size for an ensemble that includes 3 normal patterns, 2 shift patterns, one trend pattern, and one cyclic pattern in addition to the latest decision point.

Ensemble 1 is formed of 3 ANNs devoted to discovering normal patterns, 2 ANNs for shift patterns, one ANN for trend patterns, and one ANN for cyclic patterns. The last decision point is added as an input as well as the output of the selected ANNs to the leader net. If the output of the leader net is greater than an assigned threshold, the pattern is classified as a shift pattern and the process is stopped. The ARL is then the number of prior decision points. The results obtained from the ensemble are shown in Figure 15. The ARL increases as you select a higher threshold. In general, the performance of this suggested ensemble is not as good as the X-bar chart. For example, the length of the ARL at zero shift size (normal) is shorter than that of the benchmark X-bar 3 sigma which is 373.

#### 5.3.2. Ensemble 2 Results

**Figure 16:**
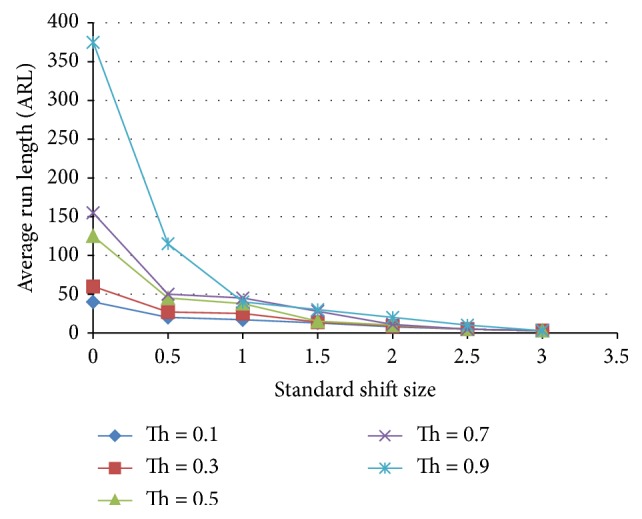
Effect of threshold on the average run length as a function of the standardized shift size for an ensemble that includes 3 normal detecting patterns, 3 shift detecting patterns, 1 cyclic pattern, and 1 trend pattern.

Figure 16 shows the average run length versus standard shift size for an ensemble with 3 ANN normal detecting patterns, 3 ANN shift detecting patterns, 1 ANN detecting cyclic pattern, and 1 ANN detecting trend pattern. The results of this ensemble are slightly better than those for the X-bar chart 3-sigma level for the case of threshold = 0.9. The ARL for the case of normal patter (zero shift size) is nearly the same as that of the X-bar chart but the ARL at a shift size of 0.5 is lower than that of the X-bar chart. Thus this ensemble has lower type I and II error.

#### 5.3.3. Ensemble III Results

**Figure 17:**
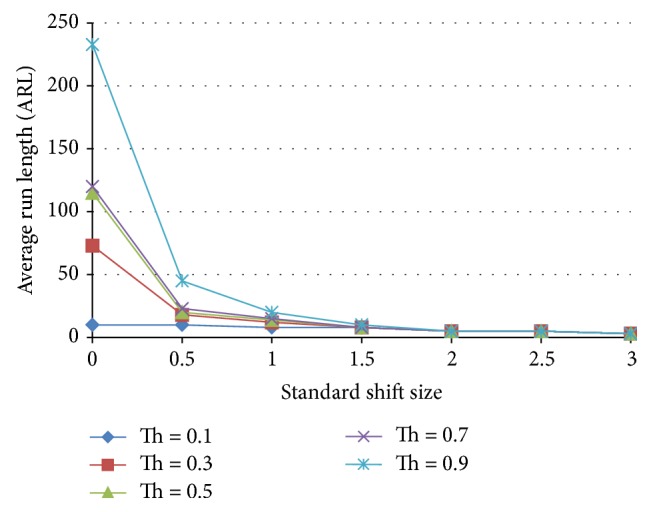
Effect of threshold on the average run length as a function of the standardized shift size for an ensemble that includes 3 normal and 3 shift ANN detecting patterns.

Figure 17 shows the results for the ensemble with 3 shift pattern detection ANNs and 3 normal detecting ANNs. The average run length of this ensemble is lower in ARL than the X-bar chart for both normal and shift behavior.

#### 5.3.4. Ensemble IV

**Figure 18:**
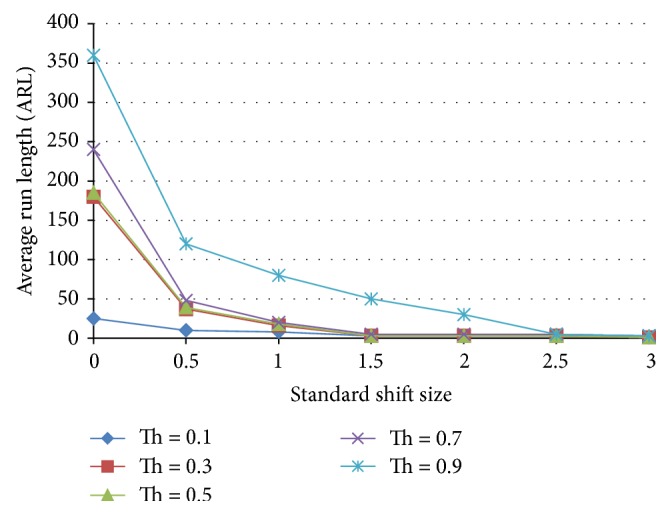
Effect of threshold on the average run length as a function of the standardized shift size for an ensemble that includes 2 shift and 2 normal pattern detecting ANNs.

Figure 18 shows the ensemble results for two normal detecting and two shift detecting neural networks. This ensemble has a behavior at threshold of 0.9 that is comparable to that of the X-bar chart.

#### 5.3.5. Ensemble V

**Figure 19:**
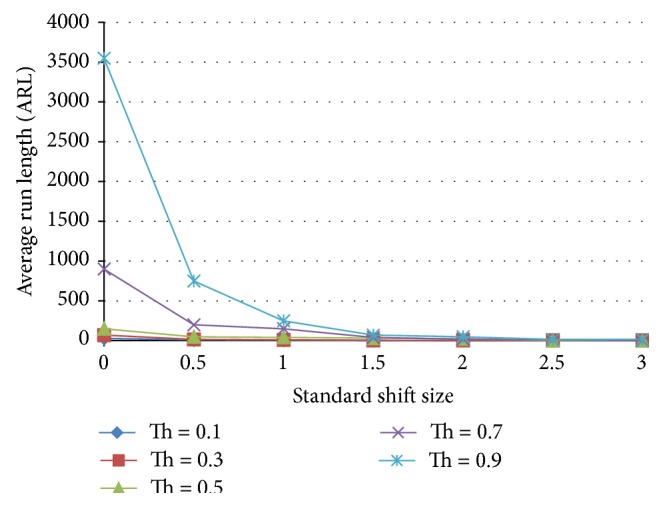
Effect of threshold on the average run length as a function of the standardized shift size for an ensemble that includes 1 normal, 1 shift, 1 trend, and 1 cyclic pattern detecting ANNs.

Figure 19 shows the ARL for 1 ANN for shift patterns, 1 ANN for normal patterns, 1 ANN for trend patterns, and 1 ANN for cyclic patterns. This ensemble includes only the best performing ANNs of each category which includes 75% normal patterns and 25% other patterns. The behavior of the ensemble at Th = 0.9 is improved for the case of normal pattern but is worse for other shift sizes. But if we closely observe the case of Th = 0.7, then we can notice that the ARL at zeroth level is increased without increasing the ARL for the case of shifted patterns.

#### 5.3.6. Ensemble VI

**Figure 20:**
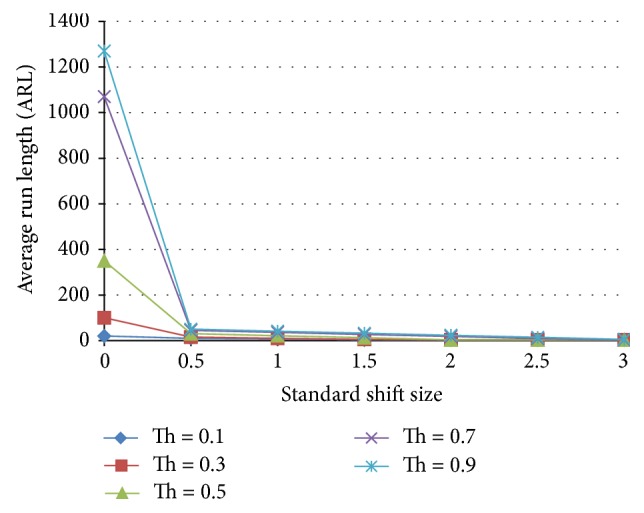
Effect of threshold on the average run length as a function of the standardized shift size for an ensemble that includes 75−25 normal and 75–25 shift pattern detecting ANNs.

Figure 20 shows the ARL results for the case of 1 normal pattern and one shift pattern which are of optimal performance. It can be noticed that, for Th = 0.9, the ARL at zero shift size is extremely higher than that of the X-bar chart while keeping the ARL for the case of shift patterns lower than those for X-bar chart and the individual chart.

## 6. Conclusions

This work represents a novel new pioneer work with high implications on fault detection. It showed that population optimised ANN ensemble can have superior performance when compared to the traditional benchmark X-bar chart. This work tested the effect of using multidecision point on the performance of the ANN, which enhanced appreciably the behavior of the ANN. This work also tested the effect of the population construction and reported that the best performance is noticed when the population construction is 75% for normal and 25% for other patterns. This work also tested several ensembles and concluded that including nonoptimized ANNs may reduce the performance of the ensemble, while including only the best performing patterns gives the best results which increases the ARL for the case of zero shift and reduces the ARL for the case of shift patterns.
